# Supplementary material for: Storable Cell-Laden Alginate Based Bioinks for 3D Biofabrication
Source: Bioengineering (Basel). 2022 Dec 23;10(1):23. doi: 10.3390/bioengineering10010023 (PMC9854877; doi:10.3390/bioengineering10010023)
Supplement: Supplementary file 1 [file bioengineering-10-00023-s001.zip › bioengineering-2028967-supplementary.pdf]

Supplementary materials

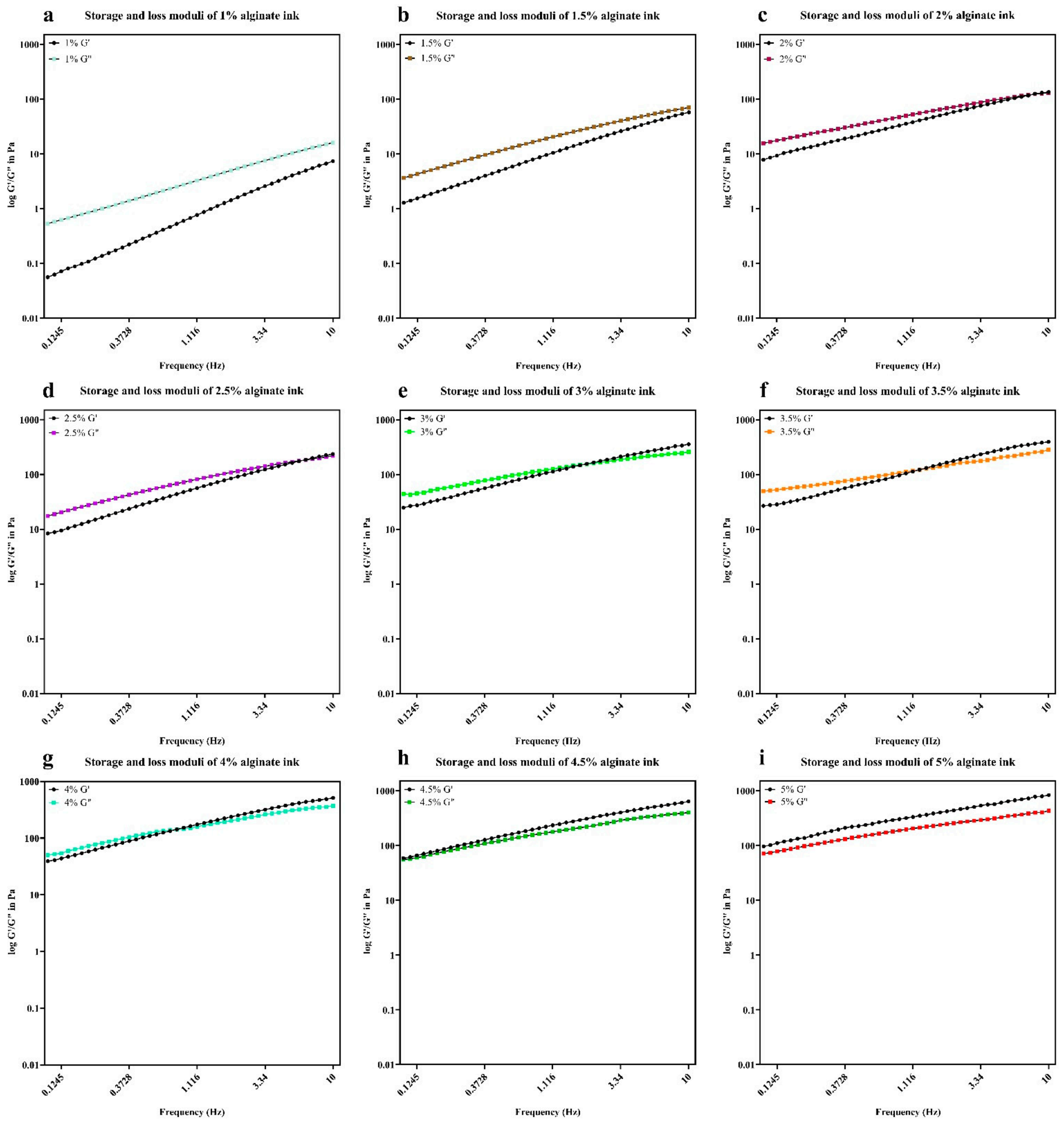

Figure S1. Storage and loss moduli of all the alginates tested. On the log y axis the storage and loss moduli of each tested alginate are displayed with shear frequency in Hz displayed on the x axis. Rheological characterization of 1% alginate is displayed in (**a**), 1.5% in (**b**), 2% in (**c**), 2.5% in (**d**), 3% in (**e**), 3.5% in (**f**), 4% in (**g**), 4.5% in (**h**) and 5% in (**i**).
